# Errata

**Published:** 1784

**Authors:** 


					and in page 320 our readers are requelted t3 rectify a miftake into
nhich we were led by the fimilarity of Chriftian names j it being, as
vre have lately been informed, not Chrifiian Frederick Lud-Migt M. D,
but his elder brother, Chrijliav Ludwig, M. D who is deceafed. He
-was born in 1743, and was the author of two Diflertations, printed at
I?eipfici.n k?73 and 1774. The firft of thefe was entitled De JE. there
yuric moto caufa diveijitatis luminum ; and the fecond, De hjUiofe ce~
rsfrri piftr-orum. ? . , : 1
E N D of V O L. V.
? i:

				

